# Application of Radiomics in Predicting the Prognosis of Medulloblastoma in Children

**DOI:** 10.3390/children12030387

**Published:** 2025-03-20

**Authors:** Jiashu Chen, Wei Yang, Zesheng Ying, Ping Yang, Yuting Liang, Chen Liang, Baojin Shang, Hong Zhang, Yingjie Cai, Xiaojiao Peng, Hailang Sun, Wenping Ma, Ming Ge

**Affiliations:** 1 Department of Neurosurgery, Beijing Children’s Hospital, Capital Medical University, National Center for Children’s Health, Beijing 100045, China; 18732612189@163.com (J.C.); dr.yangwei@foxmail.com (W.Y.); yingzesheng2022@163.com (Z.Y.); yangping5078@163.com (P.Y.); liangyuting64@126.com (Y.L.); andylc2021@163.com (C.L.); shangbj0925@163.com (B.S.); cyj04220033@126.com (Y.C.); xiaojiao428@126.com (X.P.); et2007yy@126.com (H.S.); mawenping@bjmu.edu.cn (W.M.); 2 Image Center, Beijing Children’s Hospital, Capital Medical University, National Center for Children’s Health, Beijing 100045, China; magicwind-zh@163.com

**Keywords:** medulloblastoma, radiomics features, clinical-radiomics model, prognosis

## Abstract

Background and Purpose: Medulloblastoma (MB) represents the predominant intracranial neoplasm observed in pediatric populations, characterized by a five-year survival rate ranging from 60% to 80%. Anticipating the prognostic outcome of medulloblastoma in children prior to surgical intervention holds paramount significance for informing treatment modalities effectively. Radiomics has emerged as a pervasive tool in both prognostic anticipation and therapeutic management across diverse tumor spectra. This study aims to develop a radiomics-based prediction model for the prognosis of children with MB and to validate the contribution of radiomic features in predicting the prognosis of MB when combined with clinical features. Materials and Methods: Patients diagnosed with medulloblastoma at our hospital from December 2012 to March 2022 were randomly divided into a training cohort (*n* = 40) and a test cohort (*n* = 41). Regions of interest (ROIs) were manually drawn on T1-weighted images (T1WI) along the boundary of the tumor, and radiomic features were extracted. Radiomic features related to survival prognosis were selected and used to construct a radiomics model. The patients were classified into two different risk stratifications according to the Risk-score calculated from the radiomics model. The log-rank test was used to test the difference in survival between the two stratifications to verify the classification value of the radiomics model. Clinical features related to the prognosis were used to construct a clinical model or clinical–radiomics model together with the radiomic features. Then, the clinical model, radiomics model, and clinical–radiomics model were compared to validate the improvement of radiomics in predicting the prognosis of medulloblastoma. The performance of the three models was evaluated with the C-index and the time-dependent AUC. Overall survival (OS) was defined as the time from receiving the operation to death or last follow-up. Results: A total of 81 children were included in this study. A total of five prognostic radiomic features were selected. The radiomics model could discriminate different risk hierarchies with good performance power in the training and testing datasets (training set *p*= 0.0009; test set *p* = 0.0286). Six clinical features associated with prognosis (duration of disease, risk hierarchy, dissemination, radiology, chemotherapy, and last postoperative white blood cell (WBC) level in CSF) were selected. The radiomic–clinical molecular features had better predictive value for OS (C-index = 0.860; Brier score: 0.087) than the radiomic features (C-index = 0.762; Brier score: 0.073) or clinical molecular characteristics (C-index = 0.806; Brier score: 0.092). Conclusions: Radiomic features based on T1-weighted imaging have predictive value for pediatric medulloblastoma. Radiomics has incremental value in predicting the prognosis of MB, and clinical–radiomics models have a better predictive effect than clinical models.

## 1. Introduction

MB is the most common intracranial malignant tumor in children, accounting for about 20% of central nervous system tumors in children, and the 5-year overall survival (OS) rate is 60–80% [1,2]. Patients diagnosed with medulloblastoma typically undergo stratification into high-risk and standard-risk cohorts according to their clinical features. Criteria delineating the standard-risk category in the interim encompass the absence of metastatic disease (M0 status with negative cerebrospinal fluid), age exceeding three years, and a post-surgical residual tumor volume not exceeding 1.5 cm^2^. Multiple protocols exist for the management of distinct strata of MB. The primary divergence in treatment between standard- and high-risk tumors lies in the dosage of craniospinal irradiation (CSI) administered, with standard-risk cases typically receiving 23.4 Gy compared to 36 Gy for high-risk presentations [3]. Hence, it is imperative to explore additional methodologies for discerning patients’ risk profiles prior to initiating treatment, as this facilitates informed therapeutic decision-making.

In previous studies, clinical features were used to establish a prognostic prediction model for MB [4]. It has been demonstrated that conventional MRI findings such as lesion location, morphology, enhancement pattern, and signal component characteristics have certain value in determining the pathological type and predicting the prognosis of MB. Radiomics is an emerging technique used to quantify tumor imaging features. Based on MRI, radiomics can analyze and quantify the features of the region of interest (ROI), which can provide more structural and texture information. To date, radiomic features have been used to predict the pathological classification and prognosis of MB [5,6].

This retrospective study aims to validate the value of radiomic features in stratifying the prognosis risk of MB patients. A model combining radiomic features with clinical characteristics was established to predict prognosis for MB patients.

## 2. Materials and Methods

### 2.1. Cohorts

Patients who underwent craniotomy and tumor resection and were pathologically diagnosed with MB at our hospital from December 2012 to March 2022 were enrolled. The inclusion and exclusion criteria were as follows: Inclusion criteria: (1) age 0–18 years old; (2) preoperative T1WI MRI available; (3) adequate image quality without significant artifacts; and (4) received tumor resection surgery and pathologically diagnosed with MB. Image quality was assessed by a clinical radiologist and clinical neurosurgeon, with 10 years of combined working experience. Exclusion criteria: (1) poor MRI quality and (2) lack of clinical data. Clinical characteristics (including age, sex, duration of disease, risk hierarchy, dissemination, extent of resection, ventriculoperitoneal shunt before surgery, classification, radiology, chemotherapy, preoperative blood lymphocytes, preoperative blood neutrophils, preoperative blood neutrophils to lymphocytes, preoperative blood platelets to lymphocytes, postoperative cerebrospinal fluid white blood cells) were obtained from the medical record system. Clinical data were collected according to the guidelines on the design and conduction of the first standardized database for medulloblastoma [7].

This retrospective study was carried out in accordance with the principles of the Declaration of Helsinki and approved by the Ethics Committee. Because this study was retrospective, the requirement for clinical trial registration was waived. All patients/participants provided their written informed consent to participate in this study.

### 2.2. Definition of Clinical Variables

According to the clinical data, the risk of medulloblastoma was classified. The low-risk group was defined as age > 3 years, no intracranial spread, and postoperative residual tumor < 1.5 cm^2^. The high-risk group was defined as age ≤ 3 years and/or intracranial spread and/or postoperative residue > 1.5 cm^2^. Medulloblastoma was classified into classic, desmoplastic/nodular, extensive nodular, large-cell, and anaplastic subgroups according to the pathological diagnosis. The extent of tumor resection was divided into gross total resection (GTR) and subtotal resection (STR). GTR referred to complete macroscopic resection of the tumor without radiographic residue. STR referred to the majority of the tumor being removed, but with residual macroscopic or imaging evidence remaining.

### 2.3. Experimental Design

**Figure 1:**
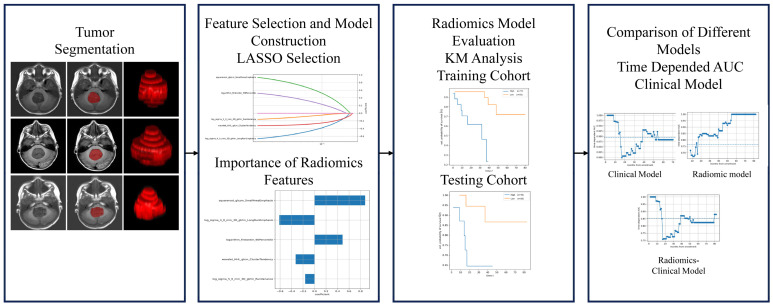
The overall design of the study.

The overall design of our study is shown in Figure 1. It consists of two parts: analyzing the predictive value of radiomic features for the OS of MB patients, and comparing the performance of the clinical model, radiomics model, and clinical–radiomics model for predicting the prognosis. In the training cohort, we first determined the most prognostic radiomic features and calculated a radiomics score (Risk-score) by using a linear combination of selected radiomic features and their weighted coefficients. Then, patients in the training cohort and testing cohort were divided into a low-risk group and a high-risk group according to the best cut-off value of the Risk-scores. The log-rank test was used to test the difference in survival between the two stratifications to verify the classification value of the radiomics model. Then, the clinical model, radiomics model, and clinical–radiomics model were constructed. The C-index and the AUC curve were used to validate the improved prediction of prognosis by radiomics.

### 2.4. MRI Imaging

All MR images were obtained during routine clinical work-up from a 3.0 T or 1.5 T scanner (General Electric, Boston, MA, USA/Philips, Best, The Netherlands) with a 12-channel receive-only head coil. Scanning parameters: T1WI: TR = 500 ms; TE = 12 ms; flip angle 70°; and slice thickness of 5 mm.

#### 2.4.1. Image Segmentation

Axial T1-weighted (T1WI) images were used for analysis. Axial T2 weighting and axial T1 enhanced weighting were used as references to plot ROIs in combination with axial T1 weighting. A total of 101 patients were recruited to participate in the experiment; 20 patients with poor imaging data quality were excluded, and a total of 81 patients were included. All the tumoral masks were independently drawn by a neurosurgeon and a neuroradiologists with more than 5 years of experience using ITK SNAP3.8.0 software (www.itk-snap.org, accessed on 15 October 2022) in the axial plane of T1WI. For discrepancies, they determined the final tumor mask through discussion.

#### 2.4.2. Radiomic Feature Extraction

The open-source Python2.7.1 tool Pyradiomics (https://pyradiomics.readthedocs.io/, accessed on 15 October 2022) was used to extract radiomic features. Shape features were extracted from the delineated ROIs to describe the shape features of the tumor. First-order gray-scale features were extracted to describe the distribution of voxel gray levels. Five different methods were used to extract texture features, including the gray-level co-occurrence matrix (GLCM), gray-level run-length matrix (GLRLM), gray-level area matrix (GLSZM), gray-level dependence matrix (GLDM), and neighborhood gray-level difference matrix (NGTDM). The wavelet transforms and four Laplacian of Gaussian (LoG) filters were used to extract the gray and texture features from the transformed images. A detailed calculation of the radiomic features can be found in the literature in the study of Zwanenburg A et al. [8]. A total of 14 shape features, 18 first-order gray-scale features, 22 GLCM features, 16 GRLM features, 16 GLSZM features, 14 GLDM features, and 5 NGTDM features were extracted from the original image. Two image filters, wavelet and Laplacian of Gaussian, were applied to the original image and produced 17 image types.

#### 2.4.3. Feature Selection

Features’ values were standardized with the z-score method. First, features with low repeatability were excluded from subsequent analysis. The Pearson correlation coefficients (PCCs) were calculated by the correlation analytical method. Any features with PCCs > 0.8 were excluded from analysis. Secondly, univariate Cox regression was used to preserve the features that were strongly correlated with survival (*p* < 0.05). Third, the importance of the remaining features was ranked, and the features whose importance was greater than 0.7 were retained. Finally, the least absolute shrinkage and selection operator (LASSO) was used to select the most relevant radiomic features. LASSO reduces the coefficients of many unrelated features precisely to zero and selects the most important features.

#### 2.4.4. Radiomics Model Construction and Evaluation

Ten-fold cross-validation was used to optimize the model, and finally, the model with the minimum cross-validation error was obtained. A radiomics score was obtained by using a linear combination of selected radiomic features and their weighted coefficients. In the training cohort, the patients were divided into low-risk and high-risk subgroups based on the cut-off value of the Risk-scores, which was determined by the Best Cut-off Analysis of the X-tile software (https://medicine.yale.edu/lab/rimm/research/software/, accessed on 15 October 2022). The cut-off was estimated on the training cohort and validated on the test cohort. The log-rank test was used to test whether the difference in survival between the high-risk and low-risk subgroups was significant. To analyze the values, *p* < 0.05 was considered significant.

#### 2.4.5. Prediction Model Construction and Evaluation

We constructed three models to further validate the improvement of radiomics in predicting the prognosis of medulloblastoma, namely a clinical model, radiomics model, and clinical–radiomics model.

Firstly, the clinical characteristics highly associated with prognosis were selected by applying Cox univariate analysis. The retained risk factors were integrated into the multivariate Cox proportional hazards model to predict outcomes and calculate the C-index and Brier score.

Then, a Cox regression model was built based on the selected radiomic features mentioned above. The C-index and Brier score were calculated to assess the performance of the Cox model.

The clinical–radiomics model was constructed by combining the selected clinical characteristics and the risk stratifications classified by the Risk-score. The risk stratifications were used as independent prognostic factors combined with clinical features to establish a prediction model, and the C-index and Brier score were calculated.

The C-index and Brier score of the three models were compared, where the higher the values, the better the results.

To further verify the incremental value of the radiomic signature over clinical molecular risk factors for individualized evaluation of OS, the time-dependent area under the curve ROC curve was calculated based on radiomic features, clinical features, and radiomic–clinical features.

#### 2.4.6. Statistical Analysis

Statistical analysis was performed using Python 3.7. The study population was characterized by descriptive parameters of mean ± SD, median (Q1, Q3), and frequency. Differences in clinical and conventional MR imaging characteristics between the training and validation cohorts were analyzed by a paired-sample *T*-test, Fisher’s exact tests, or chi-square, as appropriate. A *p* value less than 0.05 was considered statistically significant.

## 3. Results

Between July 2013 and March 2021, 101 patients underwent posterior fossa surgery at our hospital; 20 patients with poor imaging data quality were excluded, and 81 patients were included in this study. The patients were randomly divided into a training cohort (*n* = 40) and a testing cohort (*n* = 41). At the end of the last follow-up (March 2022), 19 children had died (23.5%). There were no significant differences between the training and validation cohorts in terms of age ( 6.4 vs. 6.8 *p* = 0.947), sex (male percentage: 65.9% vs. 65% *p* = 1.000), duration of disease (14.0 vs. 19.0 *p* = 0.195), risk hierarchy (low-risk percentage 51.2% vs. 60.0% *p* = 0.429), dissemination (13 vs. 21 *p* = 0.095), extent of resection (GTR percentage: 87.8% vs. 85% *p* = 0.965), ventriculoperitoneal shunt before surgery (3 vs. 8 *p* = 0.180), pathologic classification (*p* = 0.475), radiology (34 vs. 33 *p* = 1.000), chemotherapy (32 vs. 35 *p* = 0.406), preoperative blood lymphocytes (2.4 vs. 2.7 *p* = 0.430), preoperative blood neutrophils (3.4 vs. 3.7 *p* = 0.127), preoperative blood neutrophils to lymphocytes (1.2 vs. 1.6 *p* = 0.416), preoperative blood platelets to lymphocytes (110.3 vs. 122.6 *p* = 0.544), postoperative cerebrospinal fluid white blood cells (10.0 vs. 10.0 *p* = 0.443), and survival time (22.0 vs28.0 *p* = 0.600). The baselines of the 81 children with MB are shown in Table 1.

**Figure 2:**
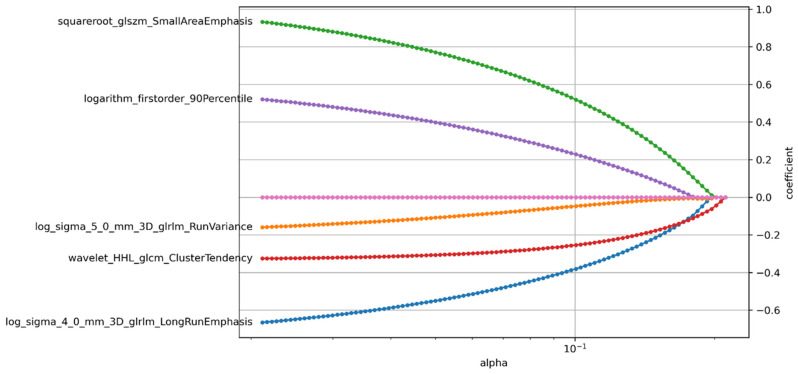
Five radiomics features with non-zero coefficients were selected by LASSO.

A total of 1561 radiomic features were extracted from the T1WI. After the correlation analysis, 56 features were retained, and then the univariate analysis selected 32 of the 56 features. After that, based on the importance ranking, the top seven features were preserved for further analysis. Finally, based on the selected features, the LASSO Cox model was built to fit the training dataset. Five radiomic features with non-zero coefficients were selected by LASSO, and a Risk-score was constructed by weighted linear combination of the coefficients of the five features (Figure 2).

The best cut-off value generated by the X-tile software was 0.41. Using this cut-off value, patients were divided into the high-risk group (Risk-score < 0.41) and the low-risk group (Risk-score > 0.41).

**Figure 3:**
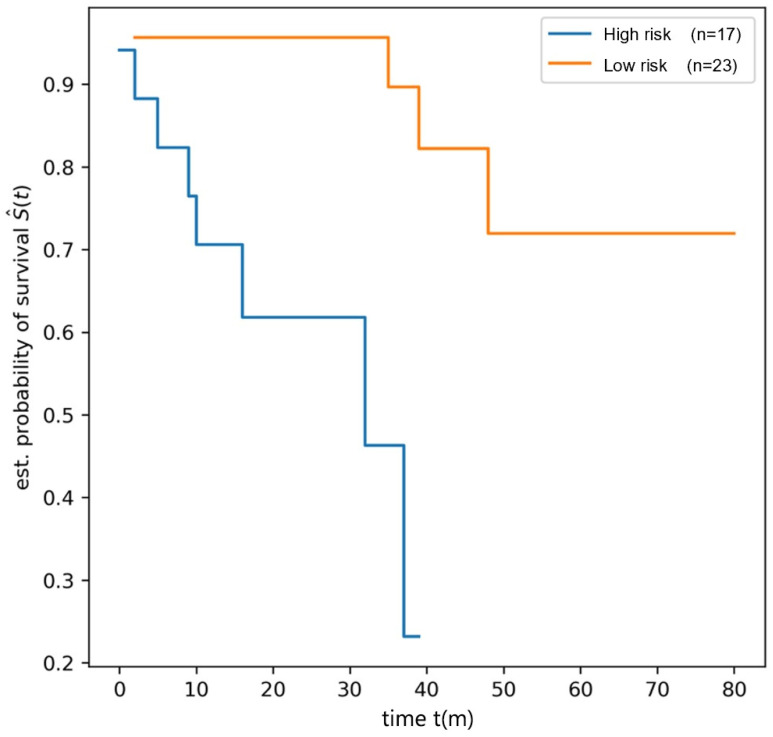
The survival curve of high-risk and low-risk groups in training dataset.

**Figure 4:**
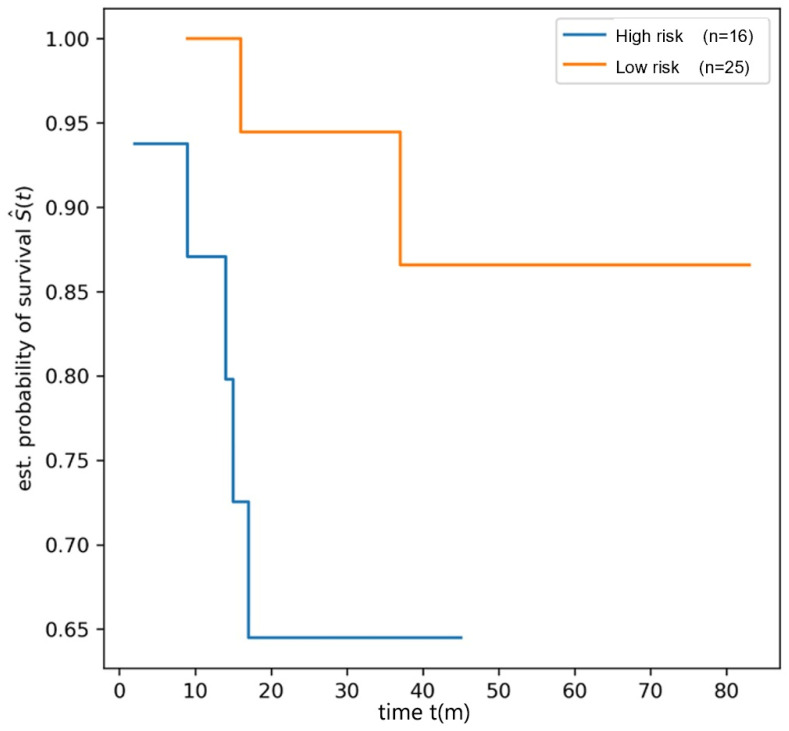
The survival curve of high-risk and low-risk groups in testing dataset.

The log-rank test detected a significant difference in OS between the high-risk and low-risk groups in the training dataset (OS 1 vs. OS 2, *p* = 0.0009, see Figure 3). In the testing dataset, the patients were stratified based on the radiomics score, and the log-rank test also demonstrated a significant difference in OS between the high-risk and low risk groups (OS 1 vs. OS 2, *p* = 0.0286, Figure 4).

**Significant clinical variables.**

The clinical model was constructed with significant clinical features in the multivariable Cox analysis. The Cox univariate regression based on clinical characteristics showed that finding-to-surgery time (HR = 1.009, *p* = 0.028), risk hierarchy (HR = 1.591, *p* = 0.001), dissemination (HR = 14.695, *p* = 0.000), radiotherapy (HR = 0.205, *p* = 0.000), chemotherapy (HR = 0.219, *p* = 0.086), and postoperative cerebrospinal fluid white blood cells (HR = 0.999, *p* = 0.019) were related to OS (Table 2).

The prediction models of OS prognosis based on clinical features, radiomic features, and clinical–radiomic features are shown in Table 2. For OS prediction, the C-index of clinical features was 0.806, and the C-index of radiomic features was 0.762. By combining the radiomics Risk-score and clinical features, the C-index of OS prognosis prediction reached 0.860 (Table 3).

**Figure 5:**
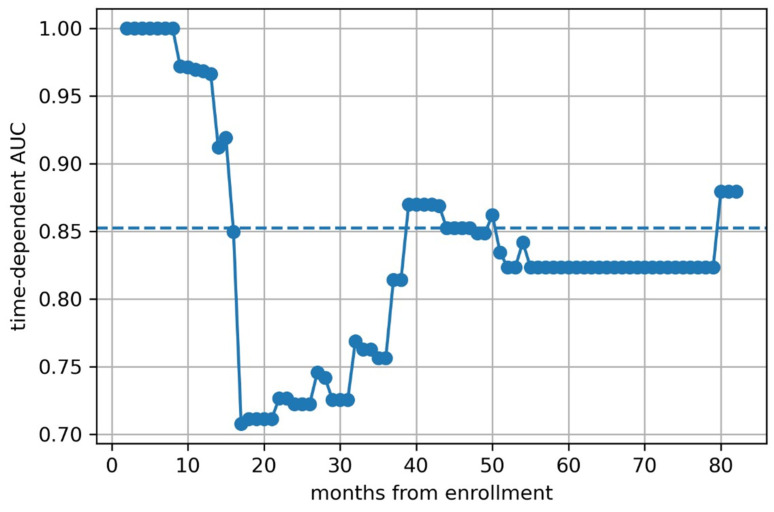
The time depended AUC of clinical model.

**Figure 6:**
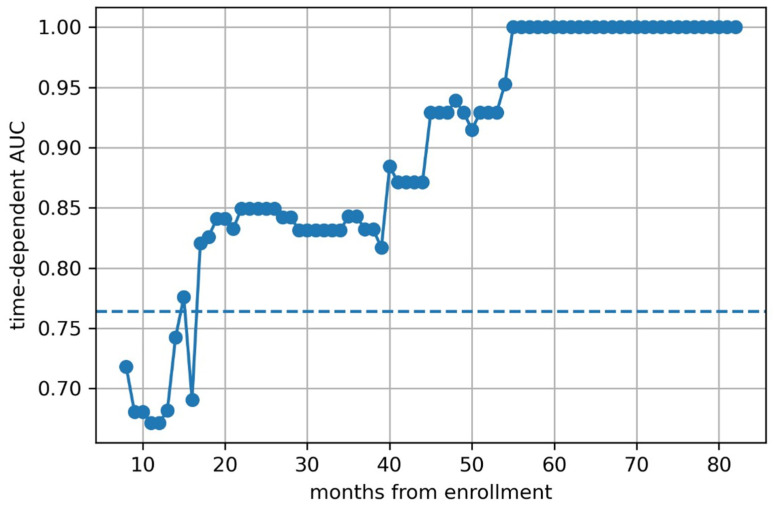
The time depended AUC of radiomic model.

**Figure 7:**
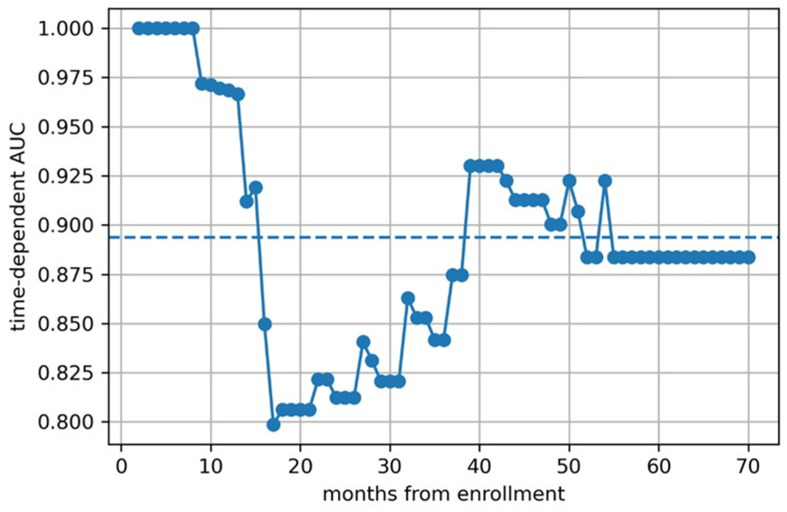
The time depended AUC of clinical-radiomic model.

We then calculated time-dependent AUCs (Figure 5, Figure 6 and Figure 7) and found that the predictive models based on clinical features and clinical–radiomic features had a greater-than-average AUC for OS prediction in the early postoperative period (≤15 months). However, in the long term (>15 months), the radiomics model had a better prognostic effect.

## 4. Discussion

In this study, we evaluated the value of MRI radiomic features for risk stratification in children with MB. The results showed that children with MB could be successfully divided into high-risk and low-risk groups based on the radiomics Risk-score, and the survival curve between the two groups showed significant differences (training set *p* = 0.0009; test set *p* = 0.0286). The accuracy of the model based on radiomics (C-index = 0.762; Brier score: 0.073) alone was lower than that of the clinical (C-index = 0.806, brier score: 0.092) model. However, the model integrating radiomic features and clinical features (C-index = 0.860; Brier score: 0.087) had better performance than the clinical and radiomics models, which implies that radiomic features derived from MRI have improvement value for survival prediction in children with MB.

Risk stratification in MB patients is currently based on clinical factors (age, presence or absence of spread, extent of excision, etc.) or molecular subgroups [4]. In the past, the age at surgery, the extent of resection, and the spread of MB were often used for risk stratification to predict the prognosis of children. Standard-risk group refers to patients > 3 years old, gross total resection (GTR), and no dissemination at the time of diagnosis. The 5-year survival rate of standard-risk children is about 70–85% [9,10,11]. High-risk group refers to patients < 3 years old, subtotal resection (STR), and/or high suspicion of dissemination. The 5-year survival rate of high-risk patients is less than 70% [10,12,13]. For the current treatment of MB, in addition to surgical resection, radiotherapy and chemotherapy play important roles in the prognosis of children. Craniospinal irradiation (CSI) is the mainstream radiotherapy for MB due to the tendency of cerebrospinal fluid dissemination in MB. Before CSI was applied, MB in older children could not be cured [14]. However, the application of CSI also brings side effects such as permanent neurocognitive disorders, neuroendocrine dysfunction, growth disorders, infertility, growth malformations, and secondary malignant tumors. In addition, CSI is not recommended for children < 3 years old, because they are in a critical period of central nervous system development. However, the role of radiotherapy in the treatment of MB is beyond doubt.

Similarly, chemotherapy has been shown to be a valuable treatment in addition to surgery and radiotherapy and has greatly improved survival in patients with metastatic and nonmetastatic MB [9,10]. In the Cox univariate regression analysis, risk stratification, radiotherapy, and chemotherapy were all highly associated with the prognosis of the children.

Many studies have found that the immune microenvironment of medulloblastoma can cause changes in white blood cells in the cerebrospinal fluid and blood. Patel et al. suggested that patients with MB may experience tumor-induced systemic immunosuppression, resulting in decreased WBC counts [15]. In contrast, some studies have found a high number of macrophages in MB patients. In addition to a significant increase in the number of macrophages, one study found that the expression of inflammatory genes corresponding to monocytes and macrophages increased in the MBSHH group. The study also reported a correlation between macrophages and proliferating tumor cells [16]. Hunter et al. found that patients who died of CNS tumors had a lower percentage of cerebrospinal fluid lymphocytes and a higher absolute cerebrospinal fluid monocyte count at diagnosis [17]. In this study, blood WBCs were included, and it was not found that the level of peripheral blood white blood cells was related to the prognosis of the patients. However, it was found that the postoperative cerebrospinal fluid white blood cell level was related to the prognosis (*p* = 0.019), which was considered to be related to the possibility of postoperative central nervous system infection. Postoperative central nervous system infection may cause a worse prognosis.

**Figure 8:**
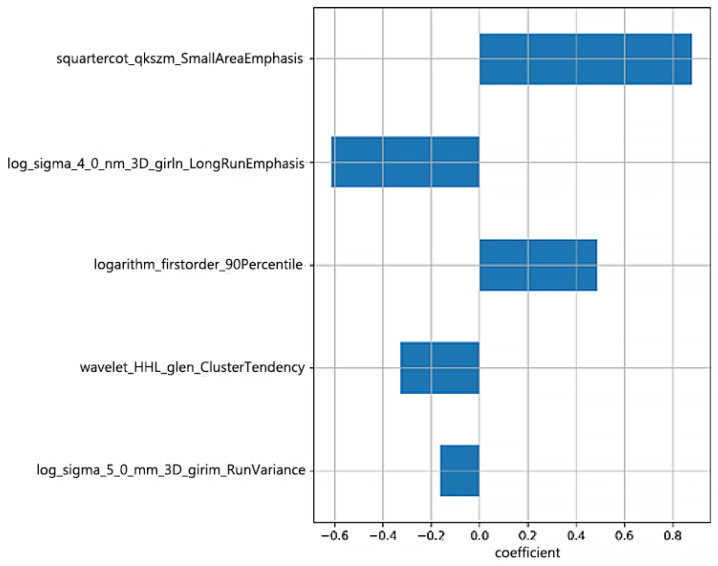
The importance of the features in the radiomics model for MB survival.

In recent years, radiomics methods have been increasingly applied to the diagnosis and treatment of MB. Rodriguez et al. included 40 children with posterior fossa tumors, extracted radiomic features from preoperative T2WI, enhanced T1WI, and ADC images, and used the support vector machine classification method to establish a model. They found that the histogram features based on ADC were the best indicators to effectively distinguish MB from PA and EP [18]. Quon et al. used deep learning to analyze the T2WI images of 617 MB and non-MB tumors and found that the accuracy of the deep learning radiomics model in differentiating pediatric posterior fossa tumors was similar to the accuracy of the readings of four experienced physicians [19]. Li et al. extracted histogram features and texture features based on the gray-level co-occurrence matrix from preoperative enhanced T1WI images of 38 cases of WNT and HH MB and found that the energy and homogeneity of the gray-level co-occurrence matrix were significantly correlated with the 2-year overall survival rates of the patients, and homogeneity was an independent factor for predicting the prognosis of WNT and SHH types [20]. In this study, based on T1-weighted imaging, 3D features, gray-scale features, and texture features of the tumor were extracted, and features highly related to prognosis were extracted after screening, as shown in Figure 8. Four of these were texture features and one was an intensity feature, which underscores the prognostic value of intensity/texture heterogeneity within the tumor for MB. In this study, we developed a radiomics model and a clinical model to assess survival. Then, we showed that an integrated model incorporating clinical–radiomic features achieved better performance and a higher C-index.

It is reasonable to infer that there is still room for improvement in existing molecular classifications and that radiomics may provide complementary prognostic information beyond molecular classifications. In the current study, radiomic features based on MR images showed favorable prognostic value in children with MB. One of the selected radiomic features comes from the first-order histogram parameter, which depends on a single pixel value and can reflect the heterogeneity of the tumor [21]. The wavelet-transformed features that can reflect the texture heterogeneity within the tumor were also selected. Some studies have shown that texture analysis based on GLCM/GLRLM can reflect the heterogeneity information of tumors [22,23]. Four prognostic radiomic texture features were found in this study, confirming that texture heterogeneity within tumors may have incremental prognostic value for MB.

Nevertheless, this study has limitations as well. First, the study sample size was not large enough (*n* = 81). Further investigations with larger sample sizes from multiple institutions are imperative to validate our findings. Secondly, molecular subtyping of MB patients was not included in our study. The molecular classification of MB is an important imaging factor for the prognosis of patients, but it was not included due to the lack of relevant data. Finally, although our study included only T1-weighted images for radiomics model construction, it is necessary to incorporate other multi-parameter MRI data into the model construction. Advanced MRI sequences such as magnetic resonance spectroscopy and dynamic sensitivity contrast (DSC) perfusion may provide more information and improve the prediction performance.

## 5. Conclusions

Radiomic features based on T1-weighted imaging have predictive value for pediatric medulloblastoma. Radiomics has incremental value in predicting the prognosis of MB, and clinical–radiomics models have a better predictive effect than clinical models.

## Figures and Tables

**Table 1:** Baselines of the MB patient cohort.

Clinical Characteristics	Testing Cohort (*n* = 41)	Training Cohort (*n* = 40)	*p* Value
Age (years)			0.947
Median	6.4 (4.1~9.6)	6.8 (4.0~9.1)	
Sex			1.000
Male	27 (65.9%)	26 (65.0%)	
Female	14 (34.1%)	14 (35.0%)	
Duration of disease (weeks)			0.195
Median	14.0 (9.0~21.0)	19.0 (11.0~25.0)	
Risk hierarchy			0.429
Low-risk	21 (51.2%)	24 (60.0%)	
High-risk	20 (48.8%)	19 (47.5%)	
Dissemination			0.095
(−)	28 (68.3%)	19 (47.5%)	
(+)	13 (31.7%)	21 (52.5%)	
Extent of resection			0.965
GTR	36 (87.8%)	34 (85.0%)	
STR	5 (12.2%)	6 (15.0%)	
V-P shunt before surgery			0.180
(−)	38 (92.7%)	32 (80.0%)	
(+)	3 (7.3%)	8 (20.0%)	
Pathologic classification			0.475
Classic	29 (70.7%)	31 (77.5%)	
Desmoplastic/nodular	2 (4.9%)	1 (2.5%)	
Extensive nodular	5 (12.2%)	7 (17.5%)	
Large-cell and anaplastic	4 (9.8%)	1 (2.5%)	
Unknown	1 (2.4%)		
Radiology			1.000
(−)	7 (17.1%)	7 (17.5%)	
(+)	34 (82.9%)	33 (82.5%)	
Chemotherapy			0.406
(−)	9 (22.0%)	5 (12.5%)	
(+)	32 (78.0%)	35 (87.5%)	
Preoperative blood lymphocytes (10^9^/L)			0.430
Median	2.4 (1.8~3.1)	2.7 (1.9~3.5)	
Preoperative blood neutrophils (10^9^/L)			0.127
Median	3.4 (2.2~5.1)	3.7 (3.0~6.9)	
Preoperative blood neutrophils to lymphocytes			0.416
Median	1.2 (0.8~2.6)	1.6 (0.9~3.7)	
Preoperative blood platelets to lymphocytes			0.544
Median	110.3 (87.3~170.9)	122.6 (85.6~193.5)	
Postoperative cerebrospinal fluid white blood cells (10^6^/L)			0.443
Median	10.0 (6.0~12.0)	10.0 (8.2~14.0)	
Survival time (months)			0.600
Median	22.0 (14.0~39.0)	28.0 (15.0~40.2)	

**Table 2:** Important factors with significant correlation with overall survival.

Factors	OS		
	HR	97.5%CI	*p*
Finding-to-surgery time	1.009	[0.969;1.052]	0.028
Risk hierarchy	1.591	[0.098;25.764]	0.001
Dissemination	14.695	[1.455;148.42]	<0.001
Radiotherapy	0.205	[0.053;0.795]	>0.001
Chemotherapy	0.219	[0.063;0.760]	0.086
Postoperative cerebrospinal fluid white blood cells	HR = 0.999	[0.996;1.002]	0.019

**Table 3:** Comparison of models’ performance.

Model	C-Index	Brier
Clinical	0.806	0.092
Radiomics	0.762	0.073
Clinical–Radiomics	0.860	0.087

## Data Availability

The data are available from the corresponding author on reasonable request.

## Abbreviations

MB, medulloblastoma; OS, overall survival; ROI, region of interest.
